# Our futile charades

**DOI:** 10.1002/jhm.13553

**Published:** 2024-11-11

**Authors:** Ella Eisinger

**Affiliations:** ^1^ Perelman School of Medicine University of Pennsylvania Philadelphia Pennsylvania USA

*Let's go down on the midaz, up on the fent*.

*First peel off the vaso, then pull back on the levo*.

*Dial down the FiO2, titrate the PEEP*.

There is so much titrating and tinkering in the ICU that at some point the patient is rendered seemingly passive, a recipient weathering the pressor escalations and opioid boluses until they meet the observational and objective parameters of comfort and clinical stability.

I think that it is because of this subliminally perceived passivity that I am so taken aback when an intubated patient breaks through the fog of sedation and begins pointing at my watch and at his mouth. Residents and I gather around his bed as the room is suddenly transformed into an enormous episode of charades in which we feverishly guess at what he is trying to say.

“It's 4:16 in the afternoon on July 22, 2024,” we say repeatedly alongside, “yes, we want to try and take that tube out soon.”

He scowls in exasperation, giving us a much‐deserved eye roll.

We try to explain that there are a few more conditions we need to optimize before he can be extubated—that he has fluid in his lungs and a new pneumonia that we are now treating, that we had tried extubation once already and wanted to offer him the best second chance possible. But he keeps pointing at his mouth and throwing his hands up in the air, his composure adopting a new flavor of attitude and rightful frustration.

“Oh, we know,” we sympathize. “We really want that tube out, too.”

He throws his hands up in the air one more time, pleading for divine intervention to knock some sense into our heads.

As I meanwhile find wonder in the emotions now alighting a face that had been rendered expressionless for days, my attending puts an end to our futile charades. She holds out a piece of paper with a grid of letters and guides a pointing tool into his hand, bridging the chasm between patient and provider.

His hand tremulously crawls across the sheet. W‐A‐T‐E‐R.

Our room falls silent, our reassurances of the imminence of extubation melting to the ground.

By my final week of my first month in the ICU as a trainee, I had come to understand how the agency of a critically ill patient is temporarily contained in favor of that same agency's long‐term preservation. Holding beneficence in the highest esteem, we ask patients to ascribe to our lab draws, treatments, and procedures; more often than not, consent is provided by surrogate decision makers in lieu of the patients themselves. And so it can provoke a sense of discomfort when a patient rouses from the sedation spell and begins to soulfully inhabit the body that until then had been rolled, stuck, and proceduralized. I wanted them to agree with the care they had been receiving, to continue along with the gameplan that we had so meticulously outlined through hours upon hours of rounds, albeit absent their direct participation. I found that any reluctance to deeply engage with the patient was more a manifestation of my own fear that they would disagree with what we had done or that we had somehow done wrong by them. And so we raced onwards toward the alliterative goals of pressor liberation, sedation vacation, extubation, and ICU graduation, suddenly caught off guard by the most vital team member's contribution.

I now know that this letter grid is a common tool for facilitating communication from critically ill patients, but I am almost embarrassed by the degree of wonder that it instilled. I am reminded of “The Diving Bell and the Butterfly,” a memoir by Jean‐Dominique Bauby who, paralyzed after a stroke, wrote the entire book by blinking his left eye when the desired letter was read aloud. In medical school we learn that a patient's history is obtained by our listening to their active speech; however, what inadvertently results is a bias toward one form of communication and a subliminal adoption of paternalism when that mode is rendered, whether temporarily or permanently, unfeasible.

I had fallen in love with the ICU early in my training because I had thought that it offered what many had described as a “new language.” In retrospect, I now recognize the drips, pressors, and vent settings instead as new vocabulary within a universally shared language of patient care. Different specialties may have different orders, note syntax, and abbreviations, but medicine's anchoring mission remains an unwavering constant.

In his book, “Every Deep Drawn Breath,” Dr. Wes Ely beautifully narrates the transformation of the culture of critical care from one of deep sedation and intubation to one that champions early rehabilitation. Through intentional sedation interruption and early mobilization, patients could be better safeguarded against long‐term cognitive and physical debility. But given that I, as a trainee, did not grow up alongside that early, appropriately challenged paradigm of critical care, I wondered why my initial schema of the specialty was premised upon it. Was it due to media depictions of critical illness or medical mores that continue to trickle down from supposedly bygone eras, surreptitiously shaping practice patterns?

His trembling hand returns to hover above the W, presumably to spell it out again given how long it had taken us to decipher his gestures in the first place. The water delivered directly to his gut to quench the neurohormonal stimuli of thirst was doing little, if anything, to soothe the dryness plaguing his mouth. Humbled, I find what is functionally a jumbo‐sized Q‐tip and swab the inside of his cheeks with water. I press it wherever I can around the endotracheal tube, for once not regarding it as the centerpiece of his problems despite its appropriate midline positioning.

Relief cascades over his face. After the frenzied gesticulations, our misinterpretations, and the patient's needs alphabetized right under our noses, it is only then that we are again speaking the same language.

## CONFLICT OF INTEREST STATEMENT

The author declares no conflict of interest.

